# Tracing leprosy trends in Pakistan: a two-decade analysis of geographic and demographic shifts (2001–2023)

**DOI:** 10.1186/s44263-025-00228-9

**Published:** 2025-12-01

**Authors:** Anil Fastenau, Nena Sophie Van Heesewijk, Matthew Willis, Paul Saunderson, Fabian Schlumberger, Ali Murtaza, Muhammed Iqbal, Abdul Salam, Sophie CW. Unterkircher, Elias Treml, Nimer Ortuño-Gutiérrez, Thomas Hambridge

**Affiliations:** 1 Marie Adelaide Leprosy Center (MALC), Karachi, Pakistan; 2 Leprosy Control Organization (LEPCO), Kabul, Afghanistan; 3 German Leprosy and Tuberculosis Relief Association (GLRA/DAHW), Würzburg, Germany; 4 Department of Global Health, Institute of Public Health and Nursing Research, University of Bremen, Bremen, Germany; 5 Heidelberg Institute of Global Health (HIGH), University of Heidelberg, Heidelberg, Germany; 6 Department of Health, Ethics and Society, Faculty of Health, Medicine and Life Sciences, Maastricht University, Maastricht, the Netherlands; 7 Hope Rises International, Greenville, SC USA; 8 Damien Foundation, Brussels, Belgium; 9 Erasmus University Medical Center, Rotterdam, The Netherlands

**Keywords:** Leprosy, Pakistan, Trend analysis, Epidemiology

## Abstract

**Background:**

Leprosy, or Hansen’s disease, is caused by *Mycobacterium leprae* and can lead to severe disabilities, social marginalisation and reduced quality of life. The disease remains a public health challenge in many low- and middle-income countries, including Pakistan. This study aimed to examine trends in leprosy cases diagnosed in Pakistan from 2001 to 2023, focusing on key epidemiological indicators such as sex, leprosy subtype, age, child cases, disability proportion and geographic distribution to reveal insights into the current situation and to inform strategies for improving case detection.

**Methods:**

This retrospective study analysed data from the Marie Adelaide Leprosy Centre (MALC), which operates 205 treatment centres across Pakistan. Leprosy cases diagnosed between 2001 and 2023 were examined for sociodemographic and clinical characteristics, including sex, age, and leprosy subtype. Descriptive statistics were presented for leprosy cases diagnosed during this period, and maps were created to illustrate geographic trends and distributions in leprosy incidence over four 5-year intervals between 2003 and 2022.

**Results:**

A total of 10,573 new leprosy cases were recorded with a median age of 36 years. Most cases (79.3%) were multibacillary (MB) leprosy. Until 2013, the majority of patients were male, but the proportion of female cases has steadily increased since then, rising from 40.4% in 2021 to 50.0% in 2023. The highest incidence was observed in Karachi, Sindh, and northern regions, including Khyber Pakhtunkhwa and Gilgit-Baltistan. The overall incidence of new cases declined steadily from 971 in 2001 to 236 in 2023. A decline in child cases and grade 2 disability proportions was also observed. Over the study period, 852 cases (8.1%) occurred in children under 15 years of age, while in 2023 specifically, 7.6% of new cases were in children and 17.4% presented with grade 2 disability, both key indicators for monitoring leprosy epidemiology. However, a substantial increase in the MB proportion was observed in 2023.

**Conclusions:**

These findings indicate significant progress in leprosy control in Pakistan, but also highlight persistent transmission in specific regions. Targeted interventions in high-burden areas, along with sustained community-based case-finding and early diagnosis efforts, are essential for continued progress toward leprosy elimination in Pakistan.

## Background

Leprosy is a chronic infectious neglected tropical disease (NTD) caused by *Mycobacterium leprae* that can lead to irreversible impairments of the skin, nerves, eyes, face, hands, and feet, as well as disability and social marginalization [1]. The disease is likely transmitted via nasal and oral secretions during prolonged and close contact with untreated patients [2]. Due to the long incubation period of *M. leprae*, which can range from months to years, leprosy incidence and transmission dynamics are difficult to estimate [3]. Although leprosy rarely directly causes death, it can indirectly reduce life expectancy. Despite the availability of effective treatment with multidrug therapy (MDT) since the 1980s, leprosy continues to persist as a public health problem in over 120 countries worldwide [1]. Around 200,000 new cases are reported annually, primarily in low- and middle-income countries, indicating the disease’s ongoing burden [1, 4].

Pakistan reached a significant milestone in leprosy control in 1996, when the disease’s prevalence fell to a point at which the World Health Organization (WHO) declared leprosy eliminated as a public health problem (prevalence < 1/10,000 population), with decreased prevalence, incidence, morbidity and mortality [5]. The country was among the first nations in the Eastern Mediterranean Region to attain this status [6]. However, control is not synonymous with elimination when new cases continue to emerge in many areas of the country [5]. Between 2005 and 2021, the annual total of new leprosy cases in Pakistan ranged from 551 in 2005 to 285 in 2021 [7]. In 2023, there were 236 new leprosy cases reported in Pakistan [8]. Notably, incidence appears to be steadily declining in recent years, with 236 new cases reported in 2023, including 121 (51.3%) female cases and 50 (21.2%) grade 2 disability (G2D) cases [9].

The geographical concentration of leprosy cases in Pakistan, particularly in Sindh province and Karachi, has drawn significant attention recently [10]. According to Ghafoor et al. [11], leprosy cases in their study cohort were geographically clustered in the south of the country, with 83% reported in Sindh and 17% in Balochistan. Karachi located in Sindh province emerged as a hotspot, accounting for 63% of total cases [12]. Given these findings, Karachi assumes a critical role in understanding leprosy dynamics within this highly affected city [10]. However, previous studies in Pakistan were limited in scale, emphasizing the urgent need for large-scale research to combat leprosy effectively [11].

This study aims to present trends of leprosy cases diagnosed in Pakistan from 2001 to 2023. This includes presenting key epidemiological indicators such as sex, leprosy subtype, child cases and disability proportion, while also taking into account geographical factors with the ultimate goal of substantially improving case detection.

## Methods

### Study population

**Fig. 1:**
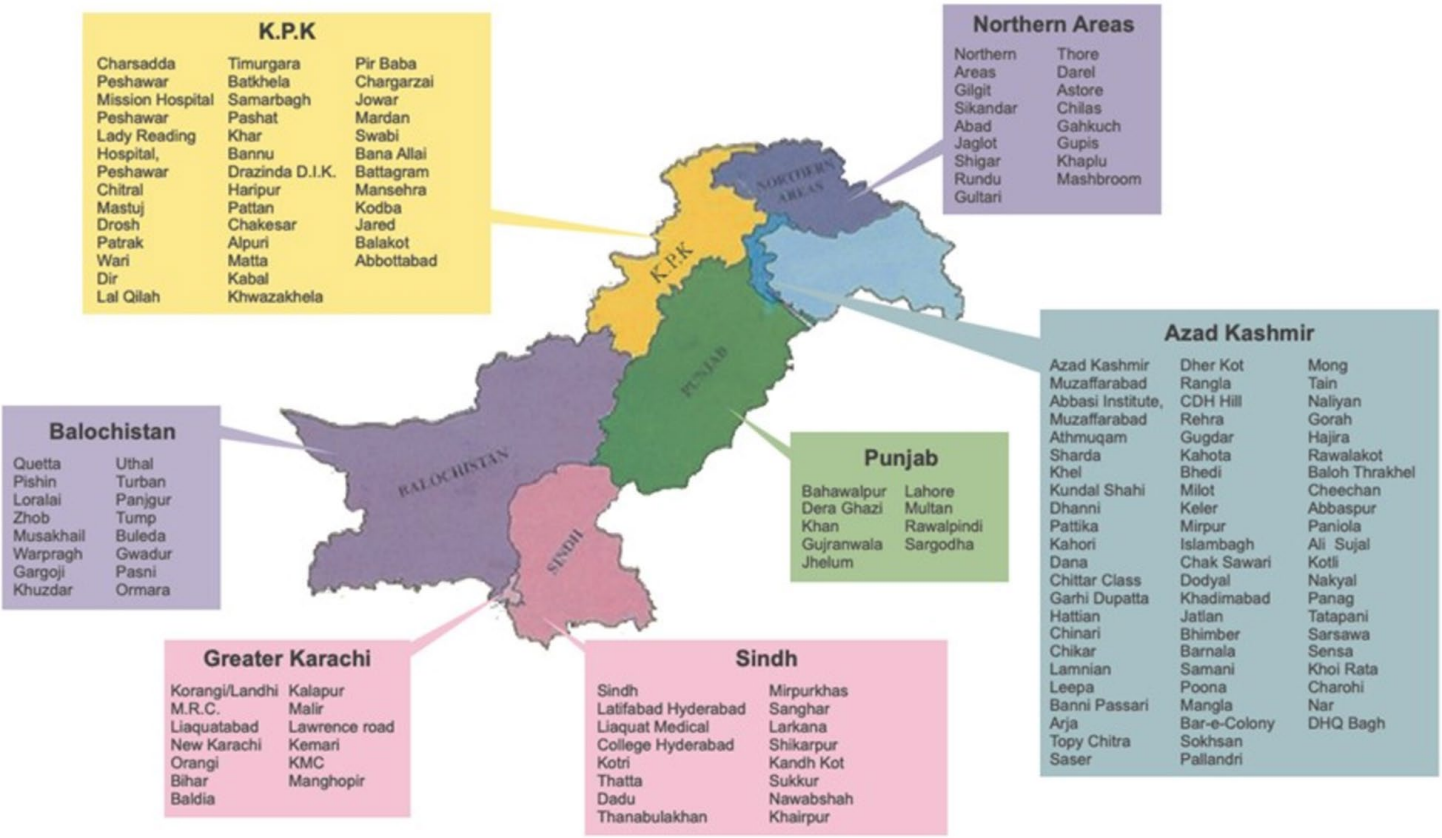
Distribution of leprosy treatment centres across Pakistan [13]. The landscape features over 205 Leprosy treatment centers, each with a Leprosy Field Officer and a devoted team of Leprosy Technicians. Their roles encompass the annual monitoring of multidrug therapy (MDT) and the comprehensive examination of all household contacts of leprosy patients

In Pakistan, leprosy control is coordinated by two NGOs that operate independently: the Marie Adelaide Leprosy Centre (MALC), based in Karachi, which covers most of the country, and Aid to Leprosy Patients (ALP), based in Rawalpindi, which is responsible for Punjab and the Hazara Division of Khyber Pakhtunkhwa; their combined statistics are reported to WHO. This study was conducted by the Marie Adelaide Leprosy Centre in Karachi, Pakistan and includes data from all 205 leprosy treatment centres across the country (Fig. 1) [13]. These centres are multipurpose units integrated into the general health system, providing not only leprosy services but also integrated skin NTD care.

The MALC is responsible for screening family members and contact tracing of leprosy patients, and partners with the health departments in Sindh, Balochistan, Khyber Pakhtunkhwa, Gilgit Baltistan, and Azad Kashmir. Additionally, the Aid of Leprosy Centre extends coverage to Punjab and the Hazara division of Khyber Pakhtunkhwa.

### Study design

This was a retrospective study, with data sourced directly from the MALC, including registered child and adult patients from 205 distinct leprosy centres across Pakistan, diagnosed between January 1, 2001 and December 31, 2023. Case detection at MALC is mostly based on active case finding, conducted through routine services, household contact tracing, and occasional skin camps. Diagnosis followed WHO clinical criteria and national guidelines [14], relying on clinical examination and slit-skin smear testing, a procedure in which small incisions are made in the skin to collect tissue fluid for microscopic examination of *Mycobacterium leprae* [14]. New leprosy cases were formally registered upon confirmation at the respective centres.

### Statistical analysis

The analysis was conducted for all confirmed leprosy cases. Diagnosis followed WHO recommended criteria and national guidelines, relying on clinical examination and slit-skin smear testing. The subtype of cases was assigned according to the WHO classification system as either paucibacillary (PB) or multibacillary (MB) leprosy [15]. According to WHO, a PB case was defined as a case of leprosy with 1–5 skin lesions, without demonstrated presence of bacilli in a skin smear. An MB case was defined as a case of leprosy with more than five skin lesions, or with nerve involvement (pure neuritis, or any number of skin lesions and neuritis), or with the demonstrated presence of bacilli in a slit-skin smear, irrespective of the number of skin lesions [16]. New case counts were calculated for each year and province. Quantitative data were analysed in SPSS version 28 and descriptive statistics presented, with figures generated in Python 3.11.0 [12, 17, 18]. To quantify the temporal change in diagnosed leprosy cases over this period, Poisson regression was used to estimate the average annual reduction in incidence and incidence rate ratio (IRR) for trends in total case counts. For trends in epidemiological indicators (child, G2D, MB and female proportion) logistic regression was used with time (year of diagnosis) as a covariate and odds ratio (OR) presented. To create retrospective map series depicting leprosy case numbers across Pakistan’s districts, Microsoft Excel and the open-source software QGIS 3.34.1 were used with a shapefile of Pakistan from the Humanitarian Data Exchange [19]. Based on 2017 population data for Pakistan from the World Bank database [20], case densities per 100,000 inhabitants were calculated in Excel for each district and time interval, then linked to district geometries in QGIS. The geometries are symbolized manually, classified by these case densities, with higher densities shown in darker shades. This spatial mapping analysis allowed for the visualization of geographic distribution and temporal shifts in leprosy burden, helping to identify high-risk districts and emerging transmission patterns.

## Results

### Trends of new leprosy cases

A total of 10,573 new adult leprosy cases were recorded between 2001 and 2023 (Table 1). There were 5866 (55.5%) males and 4706 (44.5%) females. The median age of people affected by leprosy was 36 years, and the age of leprosy cases ranged from 1 to 100 years. Over this time period, there were a total of 852 (8.1%) child cases < 15 years reported. A majority of cases presented with MB leprosy, with 8387 (79.3%) detected compared to 2184 (20.7%) with PB leprosy. The highest number of leprosy cases over this time period was found in Sindh province (53.5%), followed by Punjab (18.4%) and Khyber-Pakhtunkhwa (18.3%).

**Table 1:** Sociodemographic characteristics of new leprosy cases in Pakistan during the period 2001–2023

Characteristic	*N*	%
Age		
0–14 years	852	8.1
15–30 years	3146	29.8
31–45 years	3134	29.6
46–60 years	2337	22.1
61 and above	1104	10.4
Mean/median age	37.8/36.0	
Sex		
Male	5866	55.5
Female	4706	44.5
WHO subtype		
PB	2184	20.7
MB	8387	79.3
Disability grade		
G0D	6088	57.6
G1D	2306	21.8
G2D	2178	20.6
Province		
Sindh	5655	53.5
Balochistan	696	6.6
Punjab	1951	18.4
Azad Kashmir	283	2.7
Khyber-Pakhtunkhwa	1931	18.3
Northern Areas	52	0.5
Total cases	10,573	

*Abbreviations*: *PB* paucibacillary, *MB *multibacillary, *G0D* grade 0 disability, *G1D* grade 1 disability, *G2D* grade-2 disability, *N *number

**Fig. 2:**
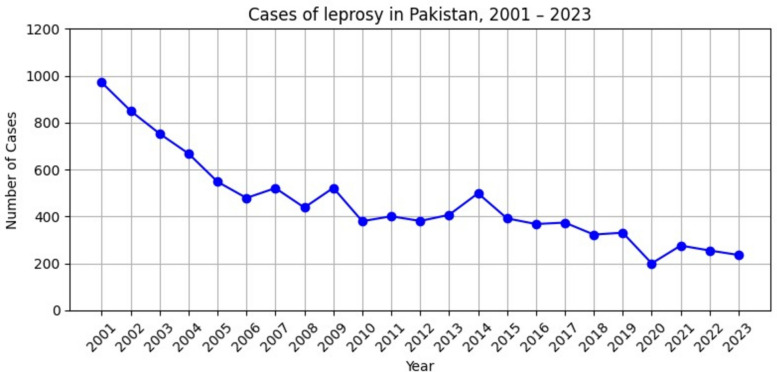
Trends in leprosy cases diagnosed in Pakistan, 2001–2023

**Fig. 3:**
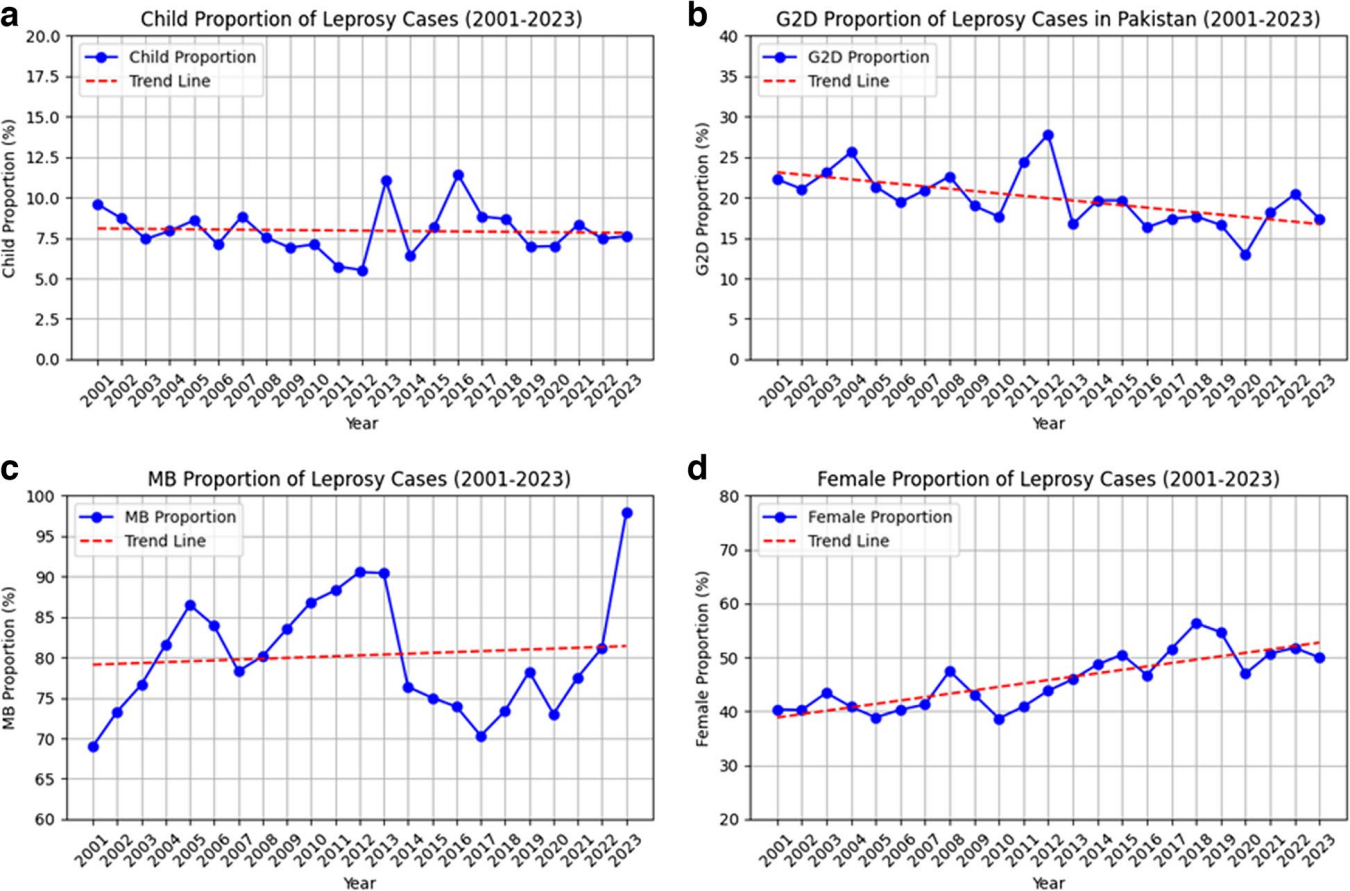
Trends in new leprosy cases in Pakistan, 2001–2023. **a** Proportion of children (< 15 years) affected by leprosy among new cases. **b** Proportion of persons with grade 2 disability among new cases. **c** Proportion of multibacillary leprosy among new cases. **d** Proportion of women affected by leprosy among new cases

There has been a clear downward trend in the total number of new leprosy cases over the past two decades, decreasing from 971 cases in 2001, to a minimum of 200 in 2020 during COVID-19, and most recently 236 cases reported in 2023 (Fig. 2), corresponding to an average 5.5% annual reduction in incidence (IRR = 0.95, 95% CI 0.94–0.95). Trends in epidemiological indicators are also presented below (Fig. 3). The proportion of new child cases (< 15 years) has fluctuated since 2010, with 18 (7.6%) reported in 2023, which has been more or less stable the past 5 years. Moreover, logistic regression analysis (Table 2) showed no significant change in the proportion of child cases over this period (OR = 1.00, 95% CI 0.99–1.01). Although the absolute number of cases with grade 2 disability (G2D) declined from 216 (22.2%) in 2001 to 50 (17.4%) in 2023 (OR = 0.98, 95% CI 0.98–0.99), the proportion of new cases presenting with G2D has remained at approximately one fifth over the past two decades, indicating that early case detection continues to be a major challenge. The MB proportion has increased significantly over time (OR = 1.02, 95% CI 1.01–1.02) but has varied considerably, with a substantial increase in the past year, increasing from 81.2% in 2022 to 97.9% in 2023. The proportion of female leprosy cases has steadily increased over time, from 40.4% in 2021 to 50.0% in 2023 (OR = 1.03, 95% CI 1.02–1.03).

**Table 2:** Statistical tests for temporal trends in leprosy indicators in Pakistan during the period 2001–2023

Indicator	Annual change (IRR/OR)	95% CI	*p*-value
New leprosy cases (total)	0.95	0.94–0.95	< 0.001
Child cases	1.00	0.99–1.01	0.488
Grade 2 disability	0.98	0.98–0.99	< 0.001
Multibacillary leprosy	1.02	1.01–1.02	< 0.001
Female cases	1.03	1.02–1.03	< 0.001

### Mapping of observed leprosy cases by district

**Fig. 4:**
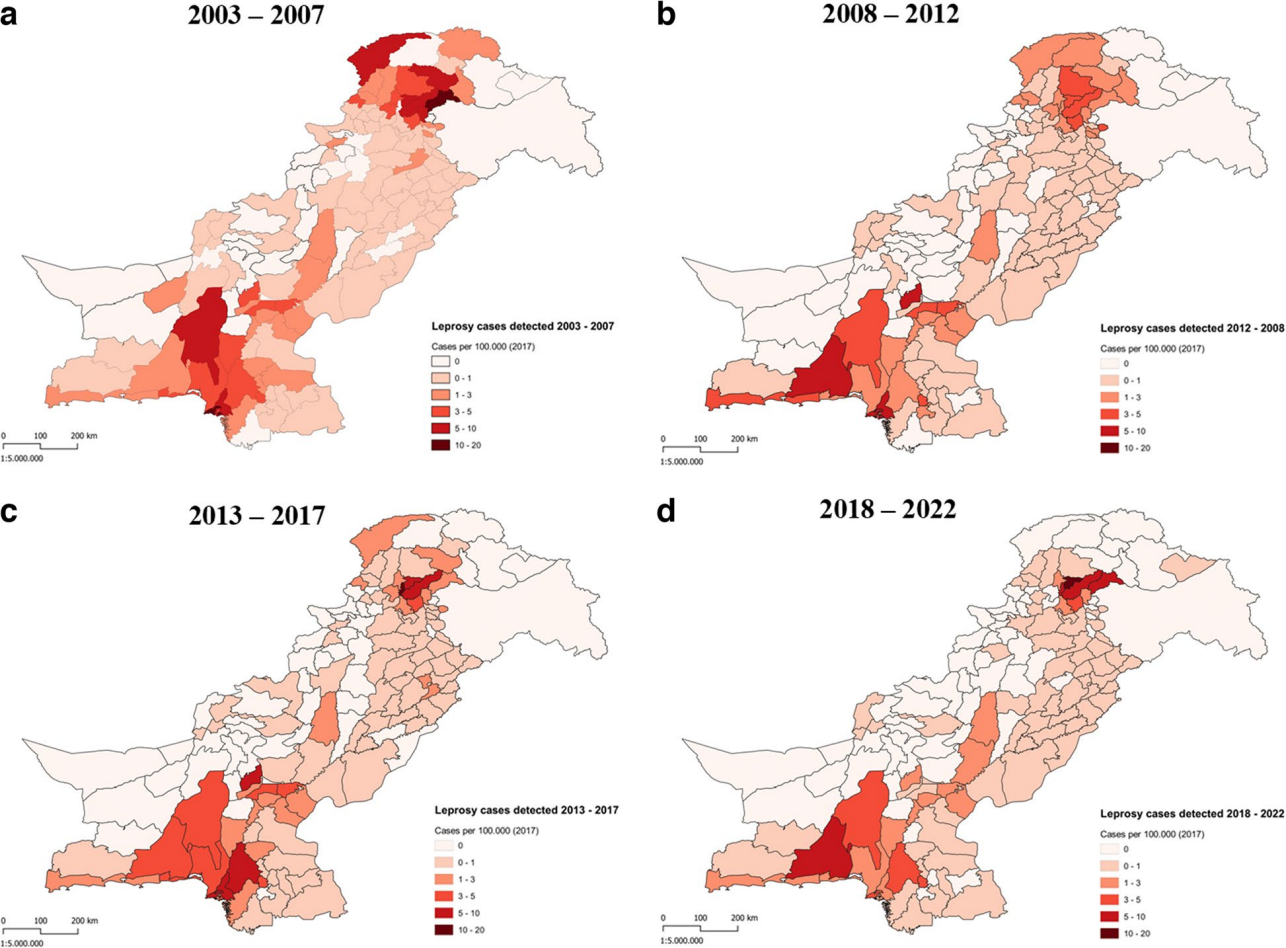
Map of new leprosy cases per 100,000 population by district in Pakistan over a 20-year period, from 2003 to 2022. Each map represents the geographical distribution of cases diagnosed within 5-year time intervals: **a** 2003–2007; **b** 2008–2012; **c** 2013–2017; **d** 2018–2022

New leprosy cases per 100,000 population in Pakistan were mapped at the district level over a 20-year period, providing a visual trend of leprosy incidence across the country and highlighting shifts in the geographic burden over time. (Fig. 4). Between 2003 and 2007, the highest number of leprosy cases was observed in the northern regions, particularly in Khyber Pakhtunkhwa and Gilgit-Baltistan, and in the southern urban centres, specifically Karachi within Sindh. Dark red areas in these regions reflect case densities exceeding 20 cases per 100,000 population. From 2008 to 2012, there was a visible shift, with sustained high case densities in Karachi and adjacent districts in southern Pakistan. Additionally, northern areas, including parts of Khyber Pakhtunkhwa and Gilgit-Baltistan, continue to show elevated case densities, though some central districts show slight reductions in incidence.

Between 2013 and 2017, a slight decline in high-density areas is observed in the northern regions, with fewer districts in Gilgit-Baltistan showing very high densities. Karachi, however, remained a consistent hotspot, maintaining persistently high case densities over time that suggest ongoing transmission and a potentially underserved population in terms of leprosy control. This pattern likely reflects a dual role of the city: on the one hand, Karachi serves as a referral hub where patients from surrounding districts are diagnosed and reported; on the other hand, transmission appears to persist within underserved urban slums. The south-central regions, including parts of Balochistan and Sindh, also continue to report moderate case densities. In the most recent 5-year period (2017–2022), high-density areas appear more restricted, with a notable decrease in the number of districts showing case densities of over 20 cases per 100,000. However, Karachi and specific districts in Gilgit-Baltistan continue to report high case density.

## Discussion

Leprosy remains a persistent but often overlooked public health issue in many countries including Pakistan, where the disease continues to affect vulnerable populations despite substantial progress in control and elimination efforts [21, 22]. The aim of this study was to present trends of leprosy cases diagnosed in Pakistan from 2001 to 2023, focusing on key epidemiological indicators such as sex, leprosy subtype, proportion of child cases and disability grade, while assessing the geographical distribution of cases. By providing insights into these trends, this analysis serves as a resource for public health professionals seeking to improve leprosy case detection and control in Pakistan. The findings demonstrate a steady decline in leprosy incidence over the two decades, especially following the WHO declaration of leprosy control in Pakistan in 1996. Despite this progress, certain regions and demographic trends reveal ongoing challenges in leprosy elimination [22, 23].

The results reveal a clear variation in the distribution of leprosy cases by region, with the highest case densities consistently observed in Karachi, Sindh, and several districts in northern Pakistan, including Khyber Pakhtunkhwa and Gilgit-Baltistan. The mapping analysis suggests that Karachi, in particular, has remained a persistent hotspot, with this urban area accounting for a substantial proportion of Pakistan’s total leprosy cases. This concentration likely reflects a combination of factors, including high population density, internal migration, and the presence of underserved urban slums where timely access to healthcare remains limited, despite the overall better availability of health services in urban areas [24]. In contrast, other regions show gradual reductions in case densities, indicating potential success in regional control efforts. These geographical disparities emphasize the need for targeted interventions that address the specific sociocultural and economic factors contributing to leprosy transmission in high-burden areas [25, 26].

The demographic trends observed in this study underscore shifts in leprosy-affected populations over time. The proportion of female cases has gradually increased, reaching parity by 2023, which may indicate that more women are now accessing diagnostic services and becoming aware of leprosy symptoms, reflecting progress in healthcare accessibility and gender equity [27]. The fluctuation in the proportion of child cases and the steady decrease in G2D proportion indicate changes in case detection and early diagnosis [28]. The declining number of new leprosy cases with G2D, specifically, reflects advancements in early detection and treatment availability, which prevent disease progression and associated disabilities [29, 30]. However, the recent increase in MB leprosy cases, particularly in the most recent year of 2023, indicates a shift toward more severe presentations. One possible explanation for the recent increase in MB cases is that intensified active case-finding activities in certain years may have led to the detection of cases that otherwise would have remained undiagnosed [8]. On the other hand, it has been observed in other settings that as the case detection rate declines over time, the characteristics of new leprosy cases tend to shift toward a higher proportion of MB cases [31, 32]. There was a noticeably sharp rise in MB proportion from 2022 to 2023, which could also reflect a change in local case finding and reporting, with leprosy detection being less emphasized and self-healing PB cases not being diagnosed. Another plausible explanation is that MB leprosy has a longer incubation period compared to PB leprosy [33]. As transmission decreases following the implementation of effective disease control programs, the number of new PB cases, which often present with subtle skin lesions, may decline more rapidly [33].

Our findings also highlight the importance of translating epidemiological trends into targeted public health actions. Strengthening systematic contact tracing and implementation of chemoprophylaxis with single-dose rifampicin (SDR-PEP) could significantly reduce transmission among household and social contacts [34]. Prioritizing high-burden areas such as Karachi and northern Pakistan for intensified interventions, including targeted community-based active case finding, skin camps, and close collaboration with local health authorities, will be crucial. Ensuring diagnostic capacity at all levels of the health system is equally important, ensuring that frontline workers remain able to recognize early signs of leprosy and other skin NTDs [23]. Improved surveillance through tools such as DHIS2 and Geographic Information System-Mapping (GIS-Mapping) can help identify transmission hotspots and monitor progress [35], while stigma reduction and community engagement will be vital to encourage early health-seeking and reduce barriers to care [8, 23]. Together, these strategies can accelerate Pakistan’s progress toward interrupting transmission and ultimately achieving zero leprosy [8].

Despite the progress, challenges remain in maintaining sustained reductions in leprosy incidence. Pakistan’s leprosy burden is closely tied to socioeconomic factors, including poverty, stigma and limited access to healthcare in certain regions, which can hinder early diagnosis and complete treatment adherence [22]. The resurgence of cases in some high-density areas underscores the necessity for continuous community-based case-finding efforts and strengthening public health infrastructure, particularly in underserved regions, such as skin camps and mobile health units [36, 37]. Collaborative efforts with community organizations, such as the Marie Adelaide Leprosy Centre, remain critical in these efforts, as they facilitate contact tracing, education, and early intervention. Future studies could focus on longitudinal tracking and genetic characterization of *M. leprae* strains to better understand transmission patterns and improve targeted interventions.

This study has several strengths, including its comprehensive analysis of a large, nationwide dataset spanning over two decades, which provides a valuable long-term perspective on leprosy trends in Pakistan. The use of data from the Marie Adelaide Leprosy Centre, one of the most extensive networks for leprosy treatment in the country, ensures high reliability in case reporting and geographical coverage [8]. Additionally, the mapping of leprosy cases by district offers critical insights into regional disparities, enabling more targeted public health strategies [35]. However, the study also has limitations. Being a retrospective study, it relies on historical records, which may be subject to reporting inconsistencies or incomplete data, particularly in more remote areas. Here, we did not investigate socioeconomic factors, such as income level or educational status, which could provide a deeper understanding of the social determinants of leprosy. Finally, the reliance on 2017 census data for case density calculations may limit the accuracy of current estimates, particularly in rapidly growing urban areas like Karachi. Future studies could address these limitations by incorporating prospective data collection and expanding socioeconomic analyses to better inform leprosy control efforts.

## Conclusions

This study highlights both progress and ongoing challenges in leprosy control in Pakistan. While there has been a clear decline in incidence and disability rates over the past two decades, certain regions and demographic groups remain disproportionately affected. Karachi and northern Pakistan, in particular, require focused public health interventions to address persistent transmission. The increasing parity in female cases and the reduction in child cases and cases with G2D are promising, while the rise in predominantly MB cases could also indicate a shift towards lower endemicity. The rising proportion of female cases aligns with a global trend [26] and may indicate improved healthcare access for women and a gradual reduction of gender inequities in leprosy detection and care, particularly in Pakistan. However, further research in Pakistan is needed to fully understand the underlying reasons for this development. Continued support for public health initiatives, particularly those that engage local communities, is essential for achieving further reductions in leprosy incidence and moving toward elimination in Pakistan.

## Data Availability

The dataset used and analysed supporting the findings of this study is retained at the Marie Adelaide Leprosy Center, Karachi, Pakistan, and cannot be made openly accessible due to ethical and privacy concerns. Data can be made available upon approval of a reasonable written request to the Chair of the MALC Ethical Review Committee: secretariat@malc.pk.

## Abbreviations

G0D: Grade 0 disability
G1D: Grade 1 disability
G2D: Grade 2 disability
IRR: Incidence rate ratio
MALC: Marie Adelaide Leprosy Centre
MB: Multibacillary
MDT: Multidrug therapy
NTD: Neglected tropical disease
OR: Odds ratio
PB: Paucibacillary
WHO: World Health Organization
